# Linear peptidomimetics as potent antagonists of *Staphylococcus aureus agr* quorum sensing

**DOI:** 10.1038/s41598-018-21951-4

**Published:** 2018-02-23

**Authors:** Georgia Karathanasi, Martin Saxtorph Bojer, Mara Baldry, Bárdur Andréson Johannessen, Sanne Wolff, Ines Greco, Mogens Kilstrup, Paul Robert Hansen, Hanne Ingmer

**Affiliations:** 1 Department of Veterinary and Animal Sciences, Faculty of Health and Medical Sciences, University of Copenhagen, Stigbøjlen 4, 1870 Frederiksberg, Denmark; 2 Department of Drug Design and Farmacology, Faculty of Health and Medical Sciences University of Copenhagen, Universitetsparken 2, 2100 København, Denmark; 3 Department of Biotechnology and Biomedicine, Metabolic Signaling and Regulation, Technical University of Denmark, Matematiktorvet, 2800 Lyngby, Denmark

## Abstract

*Staphylococcus aureus* is an important pathogen causing infections in humans and animals. Increasing problems with antimicrobial resistance has prompted the development of alternative treatment strategies, including antivirulence approaches targeting virulence regulation such as the *agr* quorum sensing system. *agr* is naturally induced by cyclic auto-inducing peptides (AIPs) binding to the AgrC receptor and cyclic peptide inhibitors have been identified competing with AIP binding to AgrC. Here, we disclose that small, linear peptidomimetics can act as specific and potent inhibitors of the *S. aureus agr* system via intercepting AIP-AgrC signal interaction at low micromolar concentrations. The corresponding linear peptide did not have this ability. This is the first report of a linear peptide-like molecule that interferes with *agr* activation by competitive binding to AgrC. Prospectively, these peptidomimetics may be valuable starting scaffolds for the development of new inhibitors of staphylococcal quorum sensing and virulence gene expression.

## Introduction

*Staphylococcus aureus* is a Gram-positive bacterium with pathogenic potential towards humans and animals alike. While considered part of the normal human flora with approximately 20–30% of healthy individuals being colonized by *S. aureus*, it is also capable of opportunistically causing a wide range of infections ranging from mild skin infections to severe conditions such as septicaemia. The ability of *S. aureus* to cause disease is attributed both to the acquisition of resistant genes (e.g., giving rise to methicillin resistant *Staphylococcus aureus*, MRSA) and to the production of an impressive collection of virulence factors^1–4^.

Expression of many of the *S. aureus* virulence factors is controlled by the accessory gene regulator (*agr*) quorum sensing system as a response to cell density^5^. The *agr* system is composed of two divergent promoters, P2 and P3. The P2 is responsible for the activation of a four-gene operon which consists of *agr*B, D, C and A. AgrD acts as precursor peptide which is synthesized in the cytoplasm and is then cleaved, modified and transported to the extracellular environment by AgrB in the form of a tailed thiolactone ring^6^. The resulting mature autoinducing peptide (AIP) consists of 7–9 amino acid residues with a five-residue thiolactone ring which is essential for its activity. AgrC and AgrA constitute a classical two component signaling (TCS) module in which AgrC is the membrane-bound receptor and AgrA is the cytoplasmic response regulator^7^. The AIP binds to the AgrC causing autophosphorylation of the cytoplasmic domain followed by transfer of the phosphate to AgrA. The phosphorylation of AgrA leads to conformational changes and subsequently, AgrA can stimulate the expression of RNAIII (the effector molecule of *agr*) via P3. The RNAIII regulation includes various virulence factor genes such as those for proteases and toxins^8^. In particular, RNAIII is responsible for the upregulation of extracellular proteins such as α-hemolysin encoded by *hla* and the downregulation of cell-surface proteins such as Protein-A encoded by *spa*. In this manner, expression of RNAIII changes the adhesion phenotype of *S. aureus* to an invasive phenotype. As the *agr* system is central to this transition, it has often been proposed as a potential target to deal with *S. aureus* infections.

Inhibition of virulence gene expression is an example of an alternative approach against *S. aureus* infections known as antivirulence therapy. This is an approach that does not affect bacterial viability and it aims to disarm the pathogen which is then expected to be killed by the host immune defense^9^. Therefore, it is believed that antivirulence therapy can pose less selective pressure to bacterial populations compared to antibiotic treatment, thus reducing the rate of resistance development to such therapeutic approaches.

Relevant to this approach, antivirulence compounds can potentially interfere with *agr* components and inhibit the expression of *S. aureus* virulence factors. An example of natural products that can target virulence gene expression through AgrC is that of Solonamide A and B which were isolated from a marine Gram-negative bacterium, *Photobacterium halotolerans*. Importantly, the chemical structures of Solonamides are remarkably similar to that of the AIP, indicating that they likely act as competitive inhibitors of AgrC-AIP binding^10^. Apart from identifying chemical entities from natural sources that act as *agr* agonists, many studies to date have also focused on the identification and/or synthesis of AIP variants in order to intercept the binding of the naturally produced AIP to the AgrC^11–15^. Protocols for non-standard chemical synthesis of AIPs have been developed^16,17^ and since *agr* interference may directly impact disease outcome^18^ and has also been elegantly demonstrated to affect bacterial behaviour on surfaces^19^ it provides attractive new routes of application within *S. aureus* antivirulence approaches.

**Figure 1:**
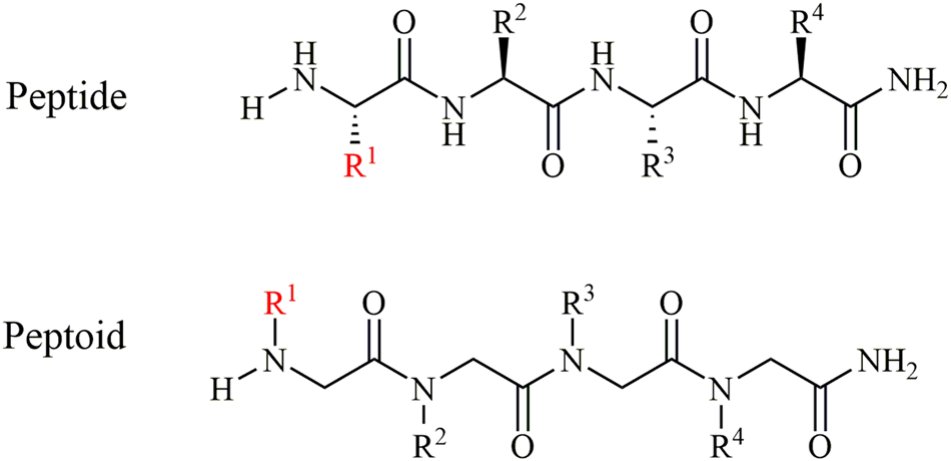
General structure of peptides and peptoids. The red color indicates one of the side chains highlighting the difference between peptides and peptoids.

Antimicrobial peptides (AMPs) have been detected in almost all living organisms including bacteria, fungi, mammals and humans as an integral part of their innate defense system^20,21^. AMPs target both Gram-positive and Gram-negative bacteria and have thus been deemed as potential candidates against bacterial infections and alternatives to antibiotics^22^. Furthermore, AMPs have been shown to display immunomodulatory activities such as leukocyte recruitment and suppression of harmful inflammation^23^. A main characteristic of AMPs is their positive charge which facilitates their interaction with the negatively charged bacterial membrane. Moreover, they exhibit a combination of hydrophilic and lipophilic properties (amphipathicity) in order to reach and penetrate the bacterial membrane through hydrophobic interactions^24^. However, one of the main disadvantages is their susceptibility to proteases. Therefore, peptide mimetics (peptidomimetics) such as peptoids (*N*-substituted oligomers of Glycine) have been proposed as improved alternatives as they retain the biological activity of the parent peptide while simultaneously displaying enhanced stability against proteases^25^. Peptoids are oligomers of *N*-substituted glycines and differ from α-peptides in that the side chains are attached to the backbone *N*^α^ position instead of the C-α atom^26^ (Fig. 1).

To date, studies have focused on the antimicrobial properties of peptoids^27–29^, including AMPs with peptoid residues in selected positions and lysine-peptoid hybrids^30–32^. These and similar compounds are often referred to as peptidomimetics^33,34^. Due to the need of compounds with novel modes of action such as compounds that can inhibit virulence gene expression, peptoids are also an interesting choice for development of new potential antivirulence candidates that may modulate the activity spectrum and/or increase the stability of traditional peptide-based AIP-AgrC antagonists. A few studies have explored the possibility of using cyclic peptoid mimics to intercept AIP-mediated *agr* activation^35,36^. To our knowledge, however, no studies have been conducted with the specific aim to assess the possibility that linear peptidomimetics could act as *agr* inhibitors. Here we examine the effects of linear peptide-peptoid hybrids on the expression of virulence factors regulated by the *agr* system in *Staphylococcus aureus* and we focus on the influence of the different side chains on antivirulence properties of these novel candidates.

## Results

**Figure 2:**
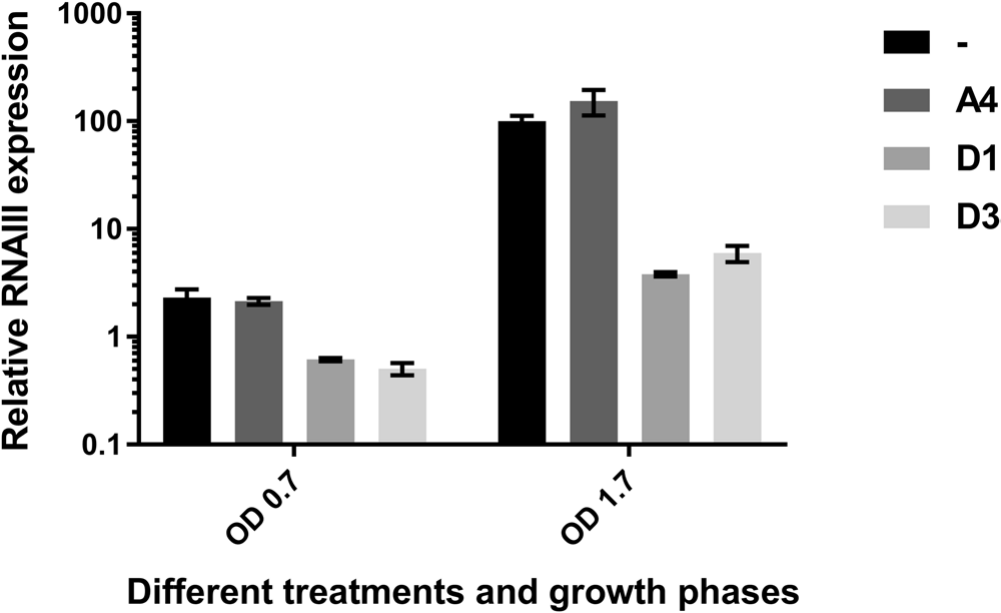
Effect of peptidomimetics on RNAIII expression. The effect of compounds A4, D1, and D3 (all 3 µg/ml) on RNAIII expression was monitored in *S. aureus* strain 8325–4 at OD_600_ 0.7 and 1.7. Each data set is based on biological duplicates with the level of the untreated culture at OD_600_ 1.7 arbitrarily set to 100(±SD).

As part of a study examining the antimicrobial activity of eight linear synthetic peptidomimetics identified from a combinatorial library, we noticed that some of these compounds could also influence virulence gene expression in *S. aureus* when applied at sub-MIC concentrations. The compounds tested were between 7 and 9 residues in length and contained L-lysine, 3-(1-naphthyl)-L-alanine (1-Nal) and the peptoid residues *N*-butylglycine, *N*-1-naphthylmethylglycine and *N*-4-methylbenzylglycine. Furthermore, the compounds carried a positive charge between 4 and 6 (Supplementary Table S1). Using a simple screen where *S. aureus* virulence gene expression is monitored in reporter strains carrying *hla*, *spa* or RNAIII promoter fusions (PC322, PC203 and SH101F7, respectively)^37^ we observed that particularly the compounds D1 and D3 and to some extent also C3 repressed *hla* and RNAIII expression while increasing *spa* expression (Supplementary Fig. S1, Supplementary Table S1). Also, when monitored by qPCR, expression of RNAIII was greatly reduced particularly in stationary phase cultures of 8325-4 (Fig. 2) that had been exposed to D1 or D3. A third compound, A4, which did not react in the plate assay screen and was included as a negative control, showed no effect on RNAIII expression thus also validating the plate assay method results. Importantly, the effect on virulence gene expression did not correlate with any antimicrobial effects as assessed from the minimal inhibitory concentrations of the compounds (Supplementary Table S2) as seen, for example, when comparing the low or non-modulatory activity of compounds C2, C4 and D4 (MICs 64–128 µg/ml) to the activity of D1 and D3 with similar MICs (128 and 64 µg/ml, respectively). These results show that linear peptidomimetics can interfere with *S. aureus* virulence gene expression and the expression pattern correlates with inhibition of the *agr* quorum sensing system.

The most active compounds identified in our screen were the D1 and D3 peptide-peptoid hybrids, which both have an *N*-1-naphthylmethylglycine and *N*-(4-methylbenzyl)glycine residue at the *N*-terminus that seems to be important for activity. A4, that lacked activity, is a peptide containing the non-proteinogenic amino acid 3-(1-naphthyl)-L-alanine and an L-lysine residue at the N-terminus. Overall, A4 has a positive charge of 6 and is more hydrophilic compared to D1 and D3, both of which carry a positive charge of 4. This is also reflected in the RP-HPLC retention times where A4 (14.8 min.) elutes earlier compared to the more hydrophobic D1and D3 (16.9 and 16.1 min. respectively).

**Figure 3:**
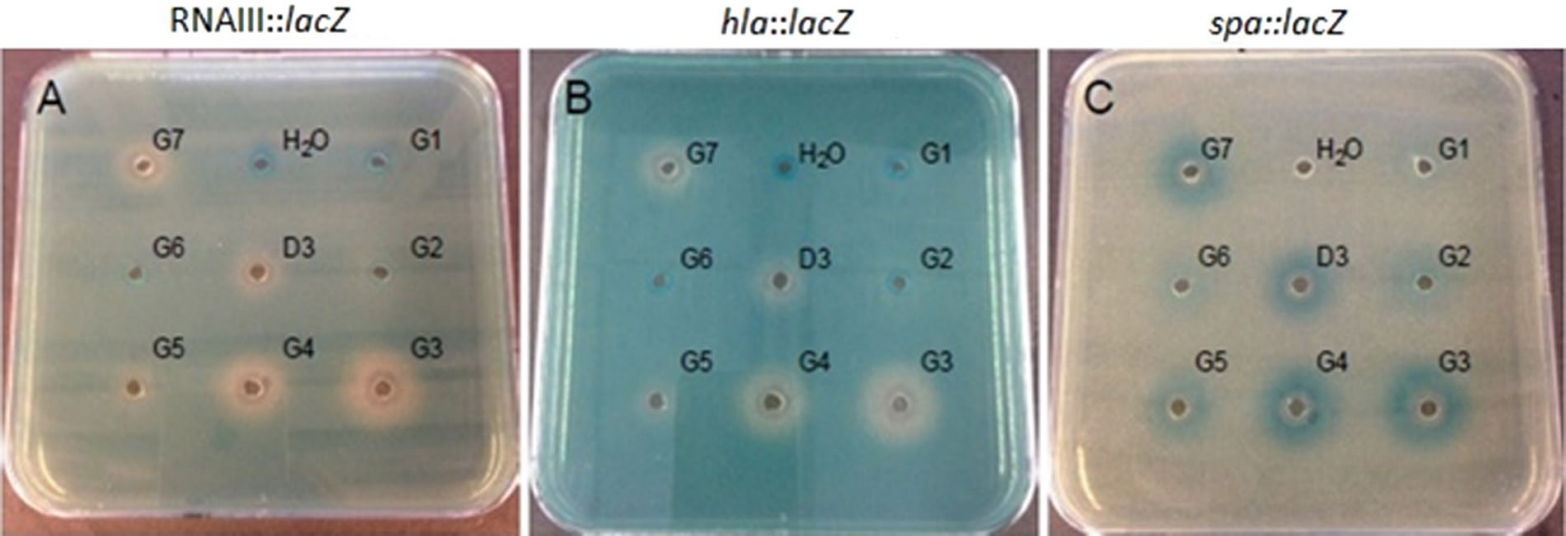
Effect of compounds G1–G7 on *S. aureus* virulence gene expression. Peptidomimetics were added (10 μg/well) to TSA plates containing SH101F7 (RNAIII::*lacZ*) (**A**), PC322 (*hla*::*lacZ*) (**B**) or PC203 (*spa*::*lacZ*) (**C**). D3 was included as reference and H_2_O was used as control.

To obtain more information about the peptidomimetic properties involved in inhibition of virulence gene expression, a glycine scan was performed^38^ in order to assess the importance of the side-chains for anti-virulence activity using D3 as a scaffold (Supplementary Table S1, G1-G7). Compound G3, in which the lysine residue in position 3 was substituted with glycine, was highly active against *S. aureus* virulence gene expresssion. This was made evident for G3 by the more pronounced downregulation of *hla* and RNAIII expression and upregulation of *spa* when compared to the other peptidomimetics tested and the parent compound D3 (Fig. 3). The derivatives G4 and G7 where lysine was replaced with a glycine residue, appeared to have increased activity as opposed to the glycine replacement of the remaining hydrophobic residues *N*-1-naphthylmethylglycine or *N*-4-methylbenzylglycine, that showed an almost complete loss of activity. Overall, the best combination in this series appears to be four hydrophobic groups, one glycine residue and two positively charged lysine residues. The balance between charge and hydrophobicity seems to be very important.This is also reflected in the HPLC retention times, where the most hydrobic compounds are G3(17.5 min.), G4(17.8 min.) and G7(17.5 min.) compared to G1, G2, G5 and G6 which have a RT between 14.4 min. and 15.7 min.

**Figure 4:**
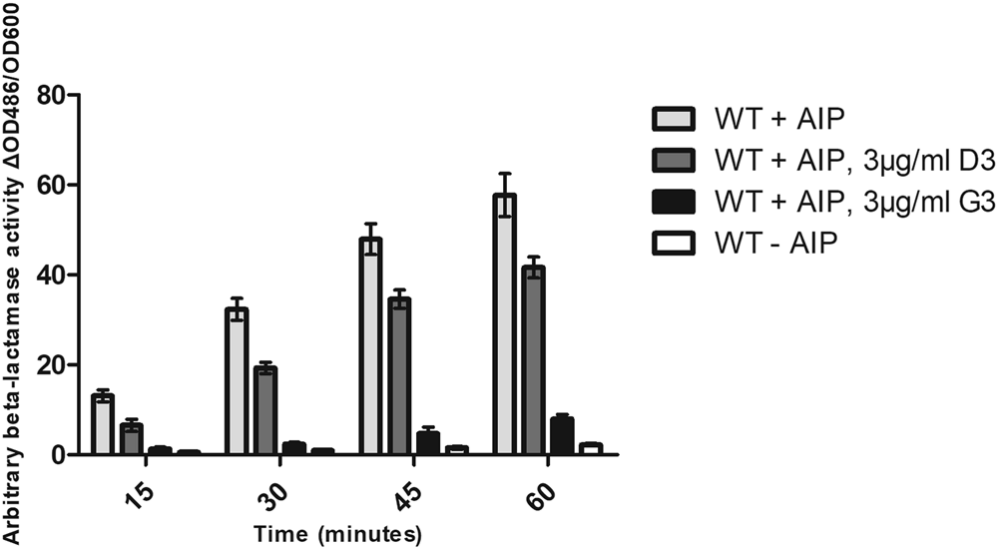
Effect of D3 and G3 on RNAIII expression in the presence of an active AgrC dependent on externally added AIP. Cultures of RN10829 WT were grown to OD600 0.4–0.5 where 10% AIP containing supernatant and 3 μg/ml compounds (final conc.) were added. DMSO was used both for the positive and the negative controls, with and without externally added AIP respectively. Bars represent the mean values (±SD) tested in biological triplicates.

The effect of G3 on RNAIII expression was confirmed in a quantitative assay using the reporter strain RN10829 WT (a derivative of 8325-4 carrying *blaZ* gene fused to the P3 RNAIII promoter)^39,40^. As this reporter strain is unable to produce AIPs, induction of *agr* and β-lactamase activity requires exogenously added AIP for instance by supplementation with spent wild type *S. aureus* supernatant. With the use of this assay we were able to confirm that G3 downregulates RNAIII expression (P3 promoter activity) to a much greater extent than the parent D3 compound (Fig. 4). Notably, the MICs of the two compounds are identical (64 µg/ml) and the pronounced effect on this alternative *agr* reporter strain at concentrations well below MIC (i.e., 3 µg/ml) further supports that the activity is related to direct *agr* interference. To evaluate *agr* inhibition on a wild type *S. aureus*, we measured RNAIII levels in the USA300-derivative strain JE2 and observed that treatment with G3 caused a 1000-fold reduction in transcript abundance upon entry into stationary phase (Supplementary Fig. S2A). The antivirulence activity of G3 was further illustrated by a strong repression of the hemolytic phenotype of *S. aureus* when grown on a blood agar plate in presence of the compound (Supplementary Fig. S2B).

**Figure 5:**
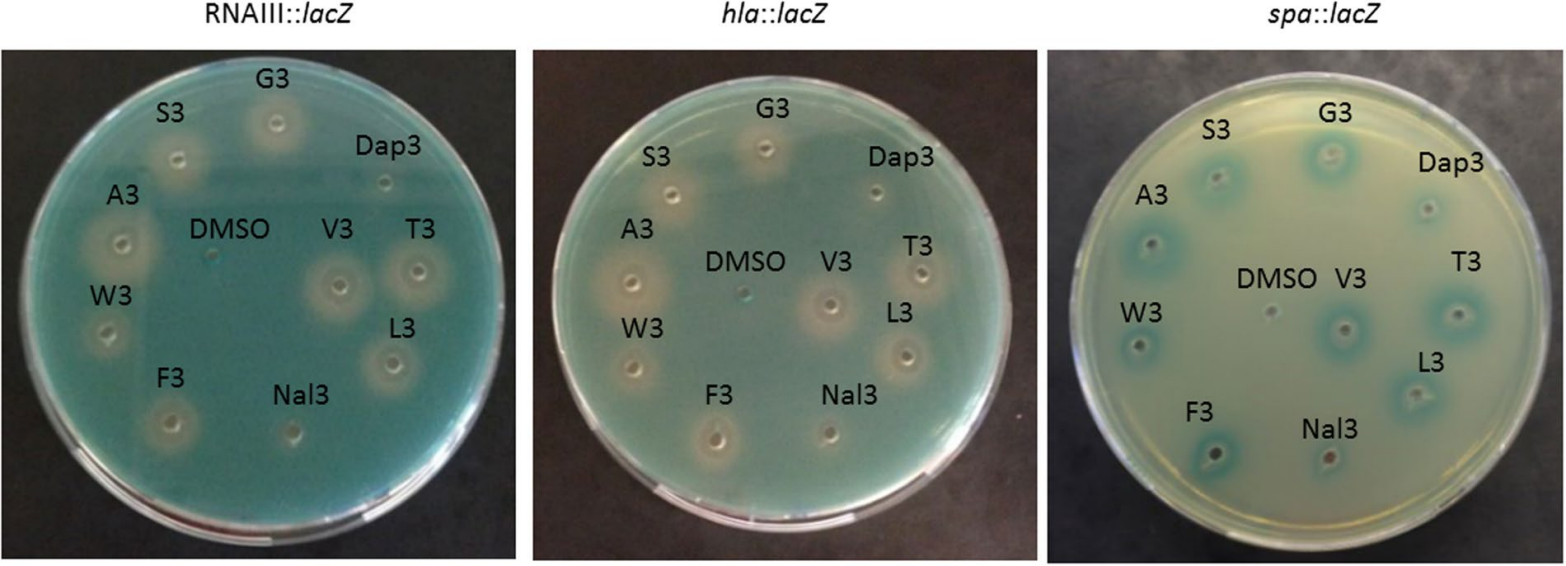
Effect of the nine D3 peptidomimetic analogues on virulence gene expression. Peptidomimetics were added (10 μg/well) to TSA plates containing SH101F7 (RNAIII::*lacZ*) (**A**), PC322 (*hla*::*lacZ*) (**B**) or PC203 (*spa*::*lacZ*) (**C**). G3 was included as reference and DMSO was used as control. Virulence gene downregulation is represented by a white zone while upregulation by a blue color zone.

**Figure 6:**
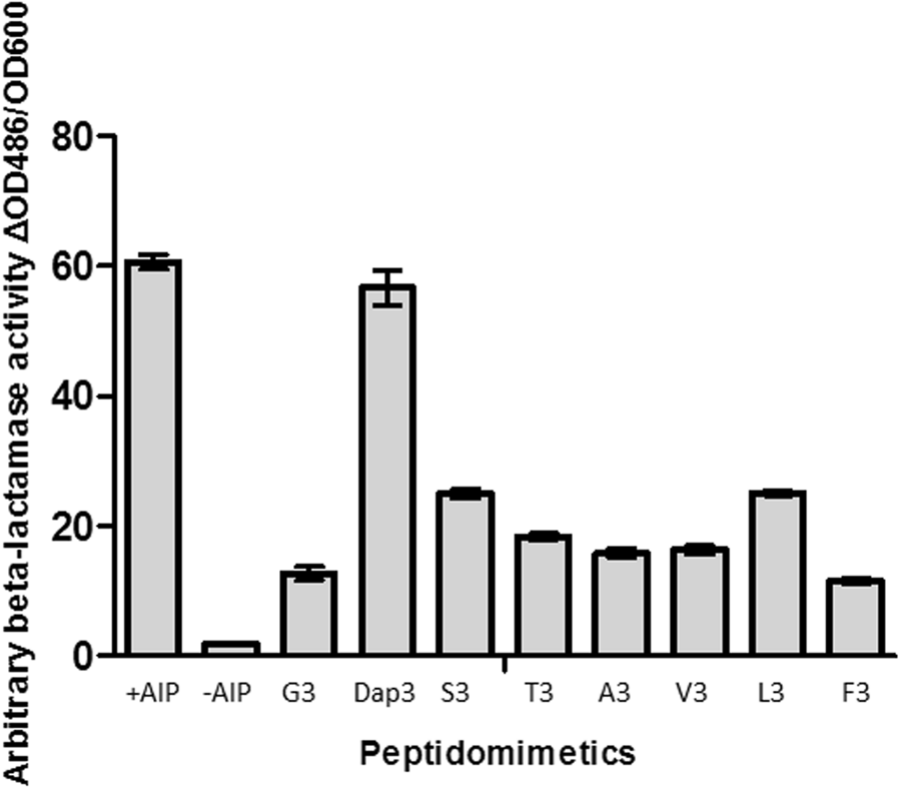
Peptidomimetics differentially influence RNAIII expression in the presence of an active AgrC dependent on externally added AIP. Cultures of RN10829 WT were grown to OD600 0.4–0.5 where 10% AIP containing supernatant and 3 μg/ml peptidomimetics were added. DMSO was used for both the positive and the negative controls which were with and without externally added AIP respectively. The data represent the mean values (±SD) from 3 individual experiments at 45 minutes after AIP addition.

It appeared that subtle modifications to the parent peptidomimetic could have important consequences regarding its anti-*agr* activity. To assess if the inhibitory activity of G3 could be enhanced even further and to assess the relevance of the chemical nature of the residue in position three, we synthesized nine new analogues where the side-chain in position three of D3 was modified by the introduction of residues with varying properties; three were cationic or polar (Dap3, S3, T3), three aliphatic (A3, V3, L3) and a further three were aromatic (W3, F3 and Nal3) (Supplementary Table S1). Screening of the nine new D3-analogues using the agar diffusion reporter assay revealed that the majority of the compounds displayed a level of activity comparable to that of G3 with the exception of Dap3, Nal3 and W3 (Fig. 5). When examined in the quantitative β-lactamase assay essentially all compounds reduced expression from the P3 promoter as did G3. In contrast, S3 and L3 exhibited a moderate reduction in activity, while Dap3 in agreement with the agar diffusion assay showed a complete loss of activity. Nal3 and W3 were excluded from further analysis due to adverse effect on the growth of the reporter strain (Fig. 6). While we were unable to further enhance the activity of G3, relative to this peptidomimetic we conclude that is possible (i) to alter the MIC of the peptidomimetic without a concomitant alteration in *agr* activity and (ii) to identify a derivative (Dap3) that is completely inactive but displays unaltered MIC. Dap3 differs from D3 in the length of aminomethyl side-chain. D3 contains a lysine residue in this position while Dap3 carries a positive charge but is three CH_2_-groups shorter, which makes it more hydrophobic (RT of 18.0 min. vs 16.1 min. for D3). For antimicrobial peptides this difference in activity is known as “the snorkel effect” which is proposes that the depth which the main-chain sinks into the lipid bilayer is determined by the length of polar residue side-chains facing away from it^41^.

**Figure 7:**
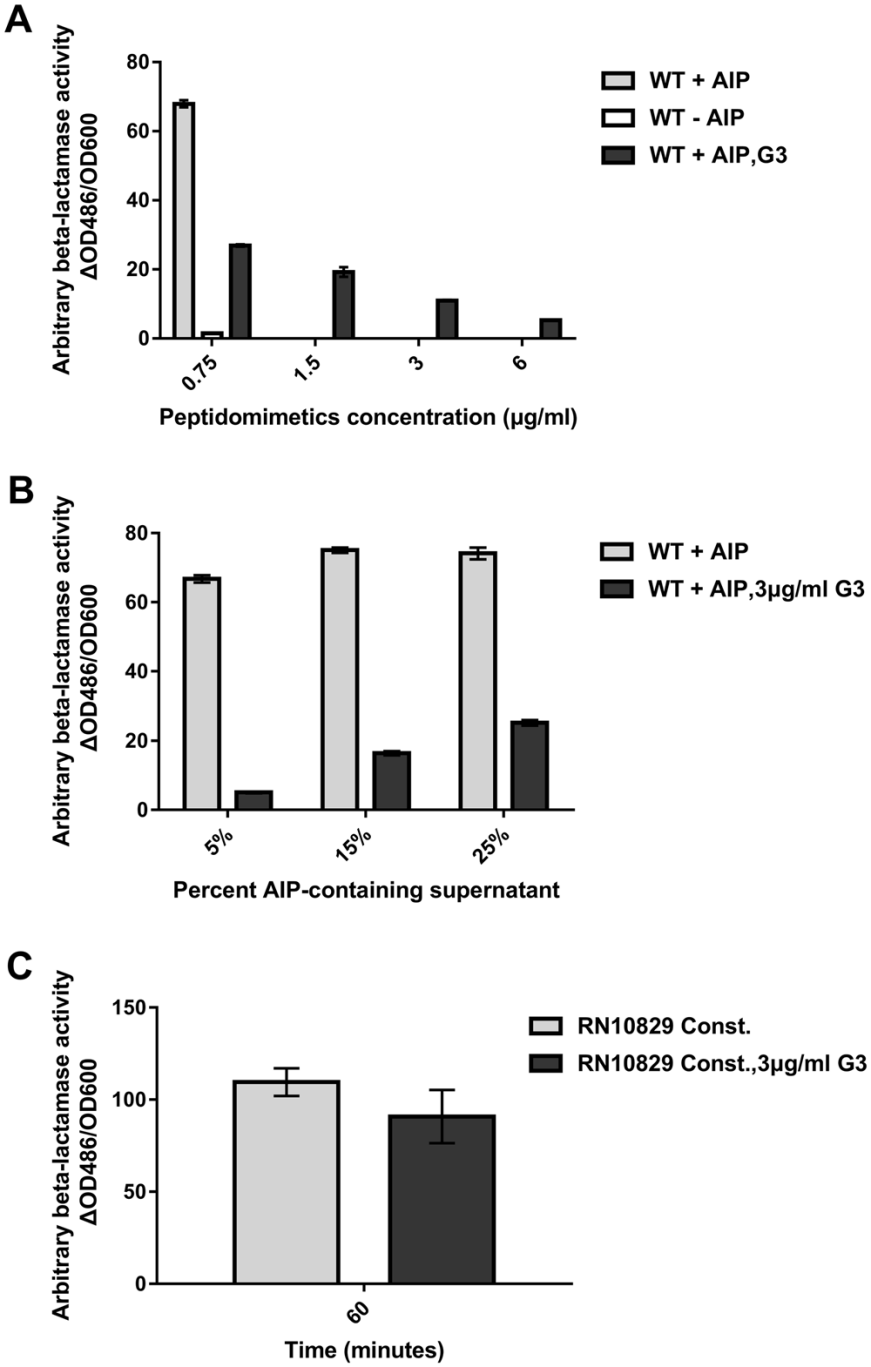
The peptidomimetic G3 acts on *agr* likely via interception of the AIP-AgrC interaction. Assessment of competitive action of G3 in a P3-*blaZ* reporter strain when applying (**A**) increasing concentrations of G3 with a fixed AIP concentration (10% AIP containing supernatant) and (**B**) increasing percentages of AIP containing supernatant with a fixed G3 concentration (3 μg/ml). The bars represent the mean values (±SD) of technical replicates of samples collected 45 minutes after addition of the AIP containing supernatant. The inhibitory effect of G3 on a strain expressing a constitutively active AgrC variant (RN10829 Const.) was assessed by treating with 3 μg/ml of the analogue (**C**) using DMSO as a control. The data represent the mean (±SD) of 3 biological triplicates of samples obtained 60 minutes after the addition of the peptidomimetic.

Having shown that peptidomimetics can interfere with virulence gene expression presumably by interfering with *agr*, we wanted to further characterize the mode of action. Over a range of sub-MIC concentrations (3–12 μg/ml) G3 did not influence growth (Supplementary Fig. S3). Next we wanted to investigate how sensitive *agr* modulation is towards varying concentrations of our peptidomimetics and AIP, as previous studies have shown that when a compound acts on *agr* via AgrC it is most likely agonist concentration dependent^40^. To address this, we monitored the antagonist action of G3 using the P3-*blaZ* reporter strain assay and observed a direct relationship between an increase in G3 concentration and the reduction in P3 promoter activity at a constant AIP concentration (Fig. 7A). Moreover, at a constant G3 concentration a gradual increase in AIP concentration led to a gradual increase in β-lactamase activity (i.e, lowering the inhibitory effect of the compound) (Fig. 7B). These results indicate that G3 (and most likely our other peptidomimetic compounds) interfere with the *agr* system through the AgrC receptor by competing with AIP-I. To further support this notion, we employed a strain with a constitutively active AgrC (RN10829 Const.)^39^ that functions independently of the externally added AIP signal. Using such a mutant strain, it is possible to assess whether any interference with the *agr* system is accomplished via or downstream of the AgrC sensor histidine kinase^42–45^. Corroborating our assumption, the data indicate that G3 acts on *agr* via AgrC as the inhibitory effect on the constitutively active AgrC reporter strain is negligible (Fig. 7C).

**Figure 8:**
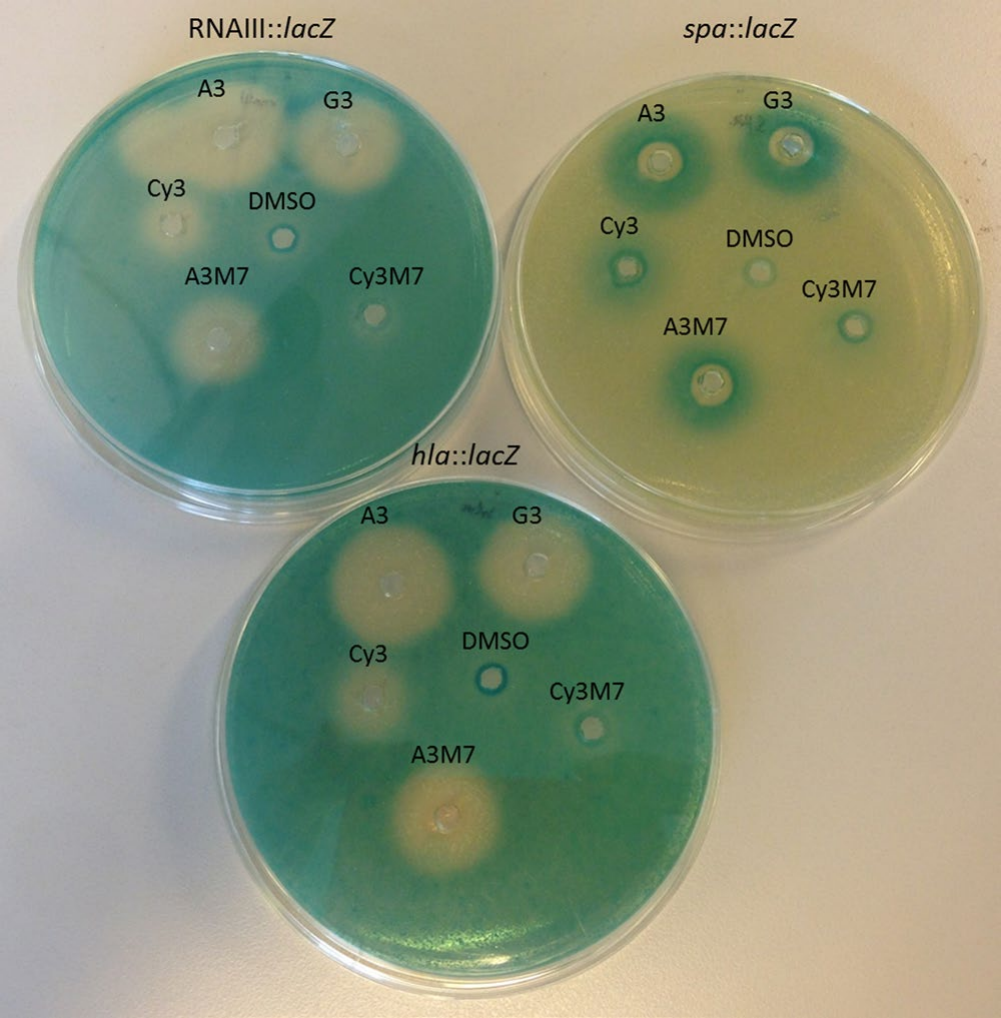
Effect of the G3, A3, Cy3, A3M7 and Cy3M7 peptidomimetics on virulence gene expression. Peptidomimetics were added (500 μg/ml; 20 μl/well) to TSA plates containing SH101F7 RNAIII::*lacZ*, PC322 *hla*::*lacZ* and PC203 *spa*::*lacZ*. DMSO was used as control. Virulence gene downregulation is represented by a white zone while upregulation by a blue color zone.

The most active peptidomimetics identified thus far (e.g. G3 and A3) are seven residues long and one could imagine that they are able to adapt a conformation which mimics AIP-I, thereby exerting their inhibitory/competitive activity. In such a scenario, residue seven might fold back proximal to residue three, thus obtaining a conformation as a five-membered pseudo-macrocyclic arrangement alike the AIP (Supplementary Fig. 4). This in turn might provide a structural explanation to the finding that G3 and G7, from the glycine scan, grouped together in terms of activity. As the macrocyclic structure of *S. aureus* AIP-I starts with a cysteine and ends with the C-terminal methionine, we aimed to test the mimicry hypothesis by synthesis of derivatives of the G3/A3 backbone with either cysteine, methionine or both appended at the relevant positions. These included Cy3 with position three of the G3/A3 backbone substituted with cysteine, A3M7 with a methionine substitution at the C-terminus of A3, and Cy3M7 containing both substitutions (Supplementary Table S1). When assayed for their *agr* modulatory activity (Fig. 8), we found that contrary to the hypothesis, neither of the single-substitutions led to a reduction in activity and the double-substitution converted the peptidomimetic into a fully inactive compound. We tested the possibility that the changes in residues had actually converted the peptidomimetics into activators of *agr* rather than inhibitors. However, we observed no induction of the P3-*blaZ* reporter by the peptidomimetics alone (not shown). Together, this suggests that the mode of action of the active peptidomimetics is not easily explained by simple AIP mimicry.

**Figure 9:**
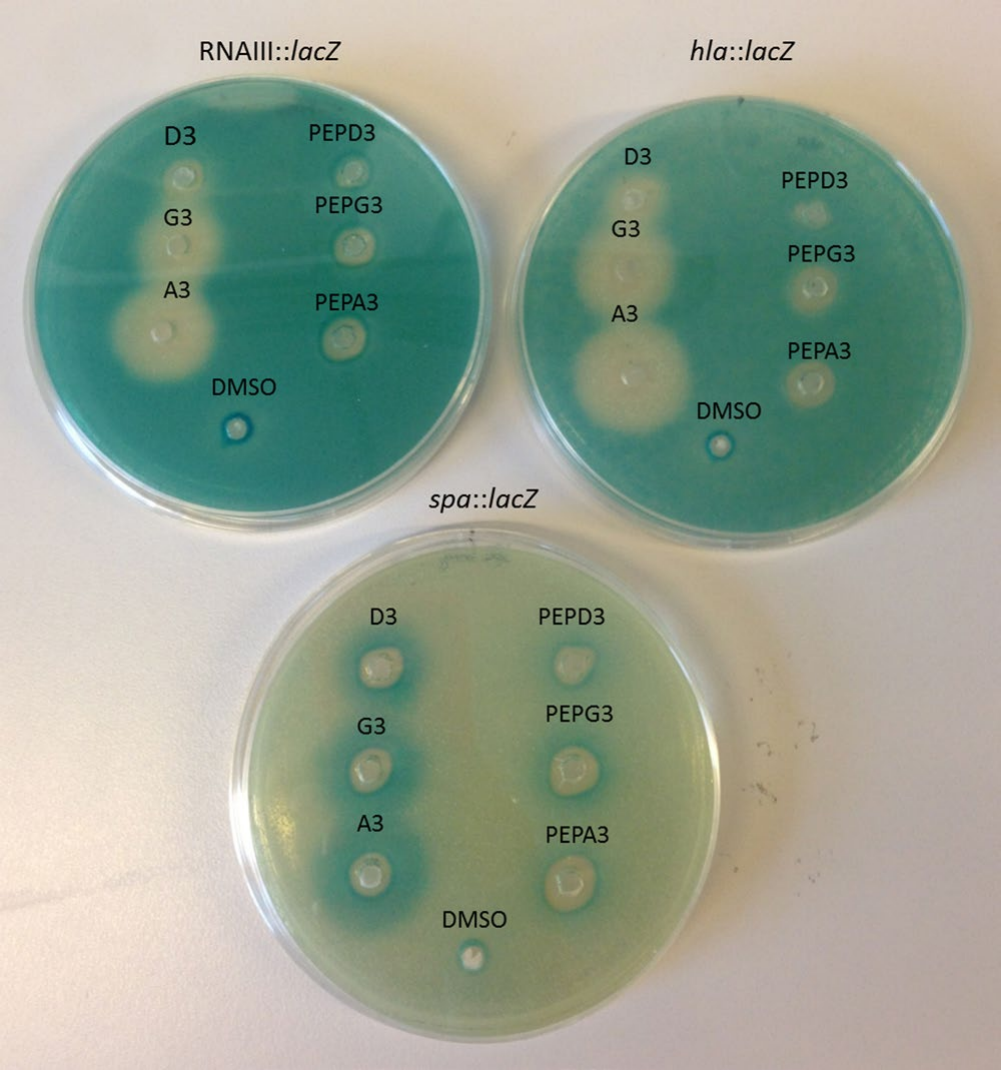
Effect of D3, G3 and A3 on the virulence gene expression in comparison with their respective peptides. Peptidomimetics and their respective peptides were added (500 μg/ml; 20 μl/well) to TSA plates containing SH101F7 RNAIII::*lacZ* (**A**), PC322 *hla*::*lacZ* (**B**) and PC203 *spa*::*lacZ* (**C**). DMSO was used as control. Virulence gene downregulation is represented by a white zone while upregulation by a blue color zone.

Finally, an important question regarding the activity of our peptidomimetics was whether their peptoidic nature is essential for their antivirulence activity or if peptide analogues could have the same activity. To address this, we synthesised the natural peptides PEPD3, PEPG3 and PEPA3 (Supplementary Table S1) corresponding to the peptidomimetics D3, G3 and A3, respectively. Strikingly, we observed that while the peptidomimetics greatly influence RNAIII, *hla and spa* expression, their corresponding peptides display essentially a complete loss of activity (Fig. 9). Hence, it is clear that the compounds need to be of a peptidomimetic nature to have *agr* modulatory activity.

## Discussion

In this study we have found that short linear synthetic peptidomimetics profoundly interfere with virulence gene expression in *S. aureus*. More specifically, these entities inhibit *agr* mediated quorum sensing and we have demonstrated that this interference likely occurs via obstructing AgrC activation by its natural AIP agonist.

When assessing the structural components important for activity we found that the G1 peptidomimetic, in which *N*-(1)-napthalenemethylamine of the parent peptidomimetic D3 was replaced with a Gly, had a remarkably reduced activity against *agr*. In contrast, G3, G4 and G7 in which Lys residues were selectively replaced with Gly showed a greatly improved *agr*-inhibitory activity compared to D3. Notably, the degree of interference displayed by the different analogues was not the same, suggesting that the position of lysine is important for their antivirulence activity. When the side-chain in position three of G3 was modified we did not further improve activity and most derivatives maintained an activity quite similar to that displayed by G3. Interestingly, however, when substituting with the small, non-proteinogenic amino acid 2,3-diaminopropionic acid (Dap) the peptidomimetic was completely inactive. This was despite the addition of the positive charge at this position, which is also a characteristic at this position in the parent compound D3. Overall, the results showed that G3 had the greatest activity, suggesting that the positive charge in position three is not important for its antivirulence activity. Importantly, the spectrum of anti-*agr* activity of our peptidomimetics appear to be completely unrelated to any antimicrobial activity and we find that the most active entities repress the quorum sensing system of our reporter strains and wild type *S. aureus* strains to a considerable degree at concentrations well below one-tenth of the MIC.

Investigation of the mode of action of G3 showed that this peptidomimetic interferes with the *agr* system by intercepting AIP mediated AgrC receptor activation. This suggests that peptidomimetics can be potential competitors of AIP, the natural ligand of the receptor. Research in this area has mainly focused on structure-activity relationships (SAR) of AIPs in order to identify compounds that repress the *agr* system in *S. aureus*^15,46^. Specifically, studies have shown that the thiolactone macrocycle is important for the inhibitory activity^13,18^; while linear variants of AIPs do not inhibit (or activate) the *agr* system^18,47^. Potent macrocycles interfering with the *agr* quorum sensing system have also been identified from nature. For example, Solonamide B which was isolated from the marine bacterium *Photobacteriumhalotolerance* acts through AgrC and has a chemical structure very similar to AIP-I^40^. Very recently, Vasquez *et al*. (2017) reported a library of 63 AIP peptidomimetics which were evaluated as AgrC receptor modulators. The authors found that three structural properties are important i) the thioester moiety since amide analogs were significantly less active ii) the presence of aromatic Phe residues on the macrocycle and iii) a macrocrocyclic ring of seven aliphatic carbon atoms appeared optimal^48^.

What makes our study interesting is that the chemical structure of our peptidomimetics differs significantly from AIPs and Solonamide; being linear and consisting of both amino acid and peptoid residues. We hypothesised that our peptidomimetics could mimic an AIP antagonist by non-covalently folding up like AIP. However, our initial efforts to synthesise derivatives with even closer resemblance to AIP-I in the primary structure did not support such a scenario as they lost rather than maintained or improved their inhibitory activity.

One of the most interesting findings in this study is that when comparing the anti-virulence activity of our best candidates (D3, G3 and A3) with their respective peptides (PEPD3, PEPG3 and PEPA3) the compounds lost their ability to repress the *agr* system. Thus, the loss of anti-virulence activity when peptoid residues are replaced by corresponding peptide residues leads to the conclusion that the peptoid structure is essential for *agr* interference. However, at present we cannot exclude an issue with instability of the peptide variants.

Collectively, this study has revealed that peptide-peptoid hybrids can be potent inhibitors of the *Staphylococcus aureus agr* quorum sensing system and that the peptoidic properties are instrumental in this activity. An advantage of our peptidomimetics compared to other compounds with a similar activity is that they are easy to synthesize due to their linear structure and thus additional complicated steps to form a macrocyclic ring are not needed. Furthermore, peptoid containing compounds are less susceptible to proteolytic degradation making them more stable than their peptide counterparts^25,49^ and studies have shown that peptoid compounds, compared to peptides, can have higher stability, higher tissue accumulation and slower elimination *in vivo*^50^. Lastly, the combinatorial flexibility in synthesis may facilitate future structure-activity relationship studies. Thus, our findings may aid future efforts into delineating essential structures needed for *agr* modulation and has brought forward a novel molecular scaffold for development of therapeutic intervention against *S. aureus* quorum sensing.

## Materials and Methods

### Synthesis of peptidomimetics

The synthesis of the peptidomimetics was performed as described by *Oddo et al*. (2015)^51^. According to this, a combination of Fmoc Solid Phase Peptide Synthesis (SPPS) and sub-monomer approach was applied. After the synthesis, the peptidomimetics were purified (>95%) by preparative HPLC and the purity was determined through analytical HPLC. Moreover, the MALDI-TOF-MS was used in order to verify the identity of the peptidomimetics^51^.

### Bacterial strains and growth conditions

Strains used in this study and their sources are listed in Table 1. Unless otherwise stated, bacteria were grown in Tryptic Soy Broth (TSB) at 37 °C with shaking at 200 rpm, with addition of antibiotics when appropriate (5 mg/l erythromycin, 10 mg/l chloramphenicol)

**Table 1:** Strains and their sources.

Strain	Description	Reference
8325–4	*S.aureus WT (agr group I)*	^53^
JE2	*Plasmid-cured version of CA-MRSA USA300*	^54^
PC203	*S. aureus* 8325-4 *spa::lacZ*	^55^
PC322	*S. aureus* 8325-4 *hla::lacZ*	^55^
RN6911	*S. aureus* (8325-4 Δ*agr)*	^56^
RN10829 WT	*S. aureus WT* *P2-agrA; P3-blaZ; pagrC-I-WT*	^39,40^
RN10829 Const.	*S. aureus (Const*.) *P2- agrA; P3-blaZ; pagrC-I-R238H (pEG11)*	^39^
SH101F7	*S. aureus* 8325-4 RNAIII*::lacZ*	^57^

### Agar Diffusion Reporter Assay

The reporter assay was conducted as described by *Nielsen et al*. (2010) using three different *lacZ* transcriptional reporter strains, PC203, PC322 and SH101F^37^. Test compounds dissolved in H_2_O or DMSO was added to agar wells at indicated concentrations and solvent only was included as a control. Incubation until blue color appeared in plates varied from 9 h to 48 h. The ability of the peptidomimetics to influence virulence gene expression was observed based on the color change around the wells; a white halo represents downregulation of virulence gene expression, while a blue halo represents upregulation of expression, with the size of the halo being indicative of the degree of interference.

### Activity of peptidomimetics in WT and Constitutive Active AgrC reporter strains using the β-lactamase assay

This assay was used as a quantitative measure of the effect of peptidomimetics on P3 promoter activity in reporter strains expressing either a wild type AgrC variant (RN10829 WT) or a constitutively active AgrC (RN10829 Const.). The method used is described by Nielsen *et al*. (2014)^40^. Briefly, 3 μg/ml of the peptidomimetics (final concentration) or DMSO (solvent), and 1/10 volume of spent medium containing or free from AIP-I were used. The β-lactamase activity of the samples was subsequently determined according to Ji *et al*. (1995) by using the nitrocefin hydrolysis method^52^.

The WT β-lactamase reporter was further employed in two modified experimental setups. To assess concentration dependence, the volume of the added AIP supernatant was kept constant (10% of the total volume) and different peptidomimetics concentrations (0.75 μg/ml, 1.5 μg/ml, 3 μg/ml and 6 μg/ml) were tested. To assess competition with AIP, peptidomimetics were added to the cell culture at the standard concentration of 3 μg/ml together with different percentages of AIP containing supernatant (5%, 15% and 25%). In both assays DMSO was used as control.

### Reverse Transcriptase-Quantitiative PCR

*S. aureus* strains (8325-4 or JE2) were grown overnight (24 h, 37 °C, 200 rpm in TSB) and diluted 100X times in fresh TSB in 100 ml Erlenmeyer flasks. The peptidomimetics were added to the flasks at a final concentration of 3 μg/ml and the cultures were incubated at 37 °C with shaking at 200 rpm. Samples were collected at OD_600_ 0.7 and 1.7, centrifuged at 4 °C, and the pellets instantly frozen at −80 °C.

RNA was purified from the frozen pellets using the Qiagen RNeasy mini-prep kit including a bead-based lysis step (FastPrep). DNA was removed with DNase I, RNase-Free (Thermo Scientific) according to the manufacturer’s instructions except for an extension of the incubation step to one hour. RNA was reverse transcribed using the High-Capacity cDNA Reverse Transcription Kit (Applied Biosystems), also including reactions containing no reverse transcriptase which were used as negative controls in subsequent amplifications. The cDNA was used as a template in the Real-Time PCR using Maxima SYBR Green qPCR Master Mix (Thermo Scientific) on a LightCycler® 96 instrument (Roche). RNAIII transcript levels were determined by the comparative cycle threshold method with *ileS* and *pyk* used for normalization applying previously published primers^11^ (Table 2).

**Table 2:** PCR primers used in this study.

Gene	Sequence Forward	Sequence Reverse
RNAIII	GCACTGAGTCCAAGGAAACTAAC	AAGCCATCCCAACTTAATAACC
*ileS*	ACATACAGCACCAGGTCACG	CGCCTTCTTCAGTAAATACACC
*pyk*	AGGTTGAACTCCCCAAACAA	GCAGCCCAAGATTACAAAAA

### Minimum Inhibitory Concentration (MIC)

Overnight cultures (24 h, 37 °C, 200 rpm in TSB) of *S. aureus* strains were diluted 5000X in fresh TSB and was aliquoted into 96-well microtiter plates with two-fold dilution series of the peptidomimetics ranging from 4 μg/ml to 256 μg/ml (final concentration) to reach an initial cell density of approximately 5 × 10^5^ CFU/ml. DMSO was used as control and the plate was incubated for 24 hours at 37 °C. The MICs of the peptidomimetics were recorded as the lowest concentration for which no bacterial growth was observed.

## Electronic supplementary material

Supplementary information
