# Supplementary material for: Linear peptidomimetics as potent antagonists of Staphylococcus aureus agr quorum sensing
Source: Sci Rep. 2018 Feb 23;8:3562. doi: 10.1038/s41598-018-21951-4 (PMC5824847; doi:10.1038/s41598-018-21951-4)
Supplement: Supplementary file 1 — Supplementary information [file 41598_2018_21951_MOESM1_ESM.docx]

**Linear peptidomimetics as potent antagonists of *Staphylococcus aureus agr* quorum sensing**

Georgia Karathanasi, Martin Saxtorph Bojer, Mara Baldry, Bárdur Andréson Johannessen, Sanne Wolff, Ines Greco, Mogens Kilstrup, Paul Robert Hansen, Hanne Ingmer

**Supplementary Information**

**Supplementary Table S1**: Chemical structures of the linear peptidomimetics derivatives with their Molecular Weight (MW).

| ID | Chemical structure | Mass calculated | Mass observed | Charge | Rt(min.) |
| --- | --- | --- | --- | --- | --- |
| A1 |  | 1056.37 | 1057.69 | +4 | 16.0 |
| A4 |  | 1312.72 | 1313.74 | +6 | 14.8 |
| C2 |  | 1198.57 | 1199.76 | +5 | 15.6 |
| C3 |  | 1070.39 | 1071.77 | +4 | 17.2 |
| C4 |  | 1198.57 | 1199.83 | +5 | 15.7 |
| D1 |  | 1070.39 | 1071.64 | +4 | 16.9 |
| D3 |  | 1070.39 | 1071.67 | +4 | 16.1 |
| D4 |  | 1198.57 | 1199.99 | +5 | 15.5 |
| G1 |  | 930.21 | 930.01 | +4 | 14.4 |
| G2 |  | 966.24 | 966.39 | +4 | 14.8 |
| G3 |  | 999.27 | 999.31 | +3 | 17.5 |
| G4 |  | 999.27 | 999.32 | +3 | 17.8 |
| G5 |  | 1014.29 | 1014.19 | +4 | 15.7 |
| G6 |  | 930.21 | 930.21 | +4 | 14.8 |
| G7 |  | 999.27 | 999.31 | +3 | 17.5 |
| Dap3 |  | 1028,31 | 1029,39 | +4 | 18.0 |
| S3 |  | 1029,30 | 1029,39 | +3 | 18.7 |
| T3 |  | 1043.32 | 1043.64 | +3 | 18.8 |
| A3 |  | 1013.30 | 1013.35 | +3 | 18.9 |
| V3 |  | 1041.35 | 1041.51 | +3 | 19.4 |
| L3 |  | 1055.38 | 1055.41 | +3 | 19.8 |
| W3 |  | 1128.43 | 1128.97 | +3 | 19.9 |
| F3 |  | 1089.40 | 1089.80 | +3 | 20.0 |
| Nal3 |  | 1139.46 | 1139.88 | +3 | 20.6 |
| Cy3 |  | 1045.34 | 1046.12 | +3 | 18.0 |
| A3M7 |  | 1016.32 | 1017.43 | +2 | 18.2 |
| Cy3M7 |  | 1048.38 | 1049.18 | +2 | 18.6 |
| PEPD3 |  | 1056.37 | 1057.69 | +4 | 15.6 |
| PEPG3 |  | 985.24 | 986.39 | +3 | 16.8 |
| PEPA3 |  | 999.27 | 1000.06 | +3 | 16.9 |

**Supplementary Table S2:** Minimum Inhibitory Concentration (MIC) of the peptidomimetics tested on 8325-4 and RN10829 WT *S. aureus* strains (MIC values were identical for both strains, thus are represented here in one column).

| **Name of analogue** | **MIC(μg/ml)** |
| --- | --- |
| A1 | >256 |
| A4 | >256 |
| C2 | 128 |
| C3 | 32-64 |
| C4 | 64-128 |
| D1 | 128 |
| D3 | 64 |
| D4 | 64-128 |
| G1 | >256 |
| G2 | >256 |
| G3 | 64 |
| G4 | 64 |
| G5 | 256 |
| G6 | >256 |
| G6 | 64 |
| Dap3 | 64 |
| S3 | 64 |
| T3 | 64 |
| A3 | 64 |
| V3 | 16 |
| L3 | 4 |
| W3 | 4 |
| F3 | 4 |
| Nal3 | 8 |

**Supplementary Figure S1**

**
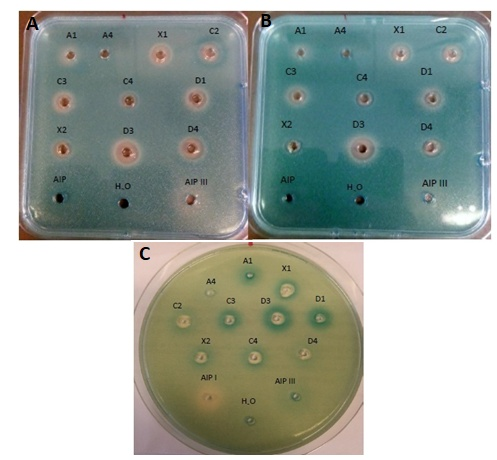
**

**Supplementary Figure S1. Effect of compounds A1-X2 on the virulence gene expression**. To TSA plates containing PC322 *hla*::*lacZ* (A), SH101F7 RNAIII::*lacZ* (B) and PC203 *spa*::*lacZ* (C) were added 20μl of the peptidomimetics (500μg/ml). Controls: AIP I, AIP III and H_2_O. Virulence gene downregulation is represented by a white zone while upregulation by a blue color zone.

C

**Supplementary Figure S2**


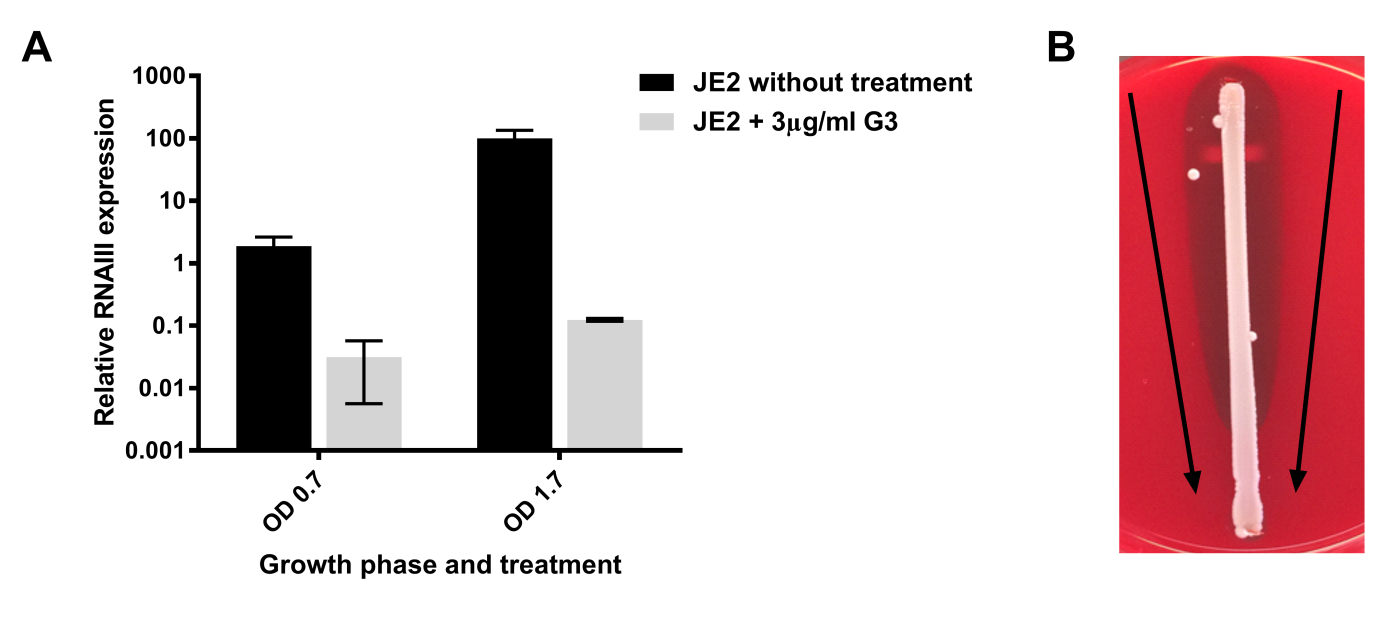


**Supplementary Figure S2. Effect of compound G3 on RNAIII and virulence expression in strain JE2.** (A) The effect of peptidomimetic G3 (3 µg/ml) on RNAIII expression was monitored in *S. aureus* strain JE2 at OD_600_ 0.7 and 1.7. The data set is based on biological triplicates with the level of the untreated culture at OD_600_ 1.7 arbitrarily set to 100 and the error bars representing the standard deviation. (B) Assessment of the effect of G3 on *S. aureus* hemolysin expression on blood agar plate. 5 µl drops of G3 (2 mg/ml) were positioned along a streak of *S. aureus* strain JE2 at decreasing distance from the bacteria (indicated by arrows). Complete repression of toxin production indicated by lack of hemolysis is evident where growth of the bacteria is unaffected.

**Supplementary Figure S3**


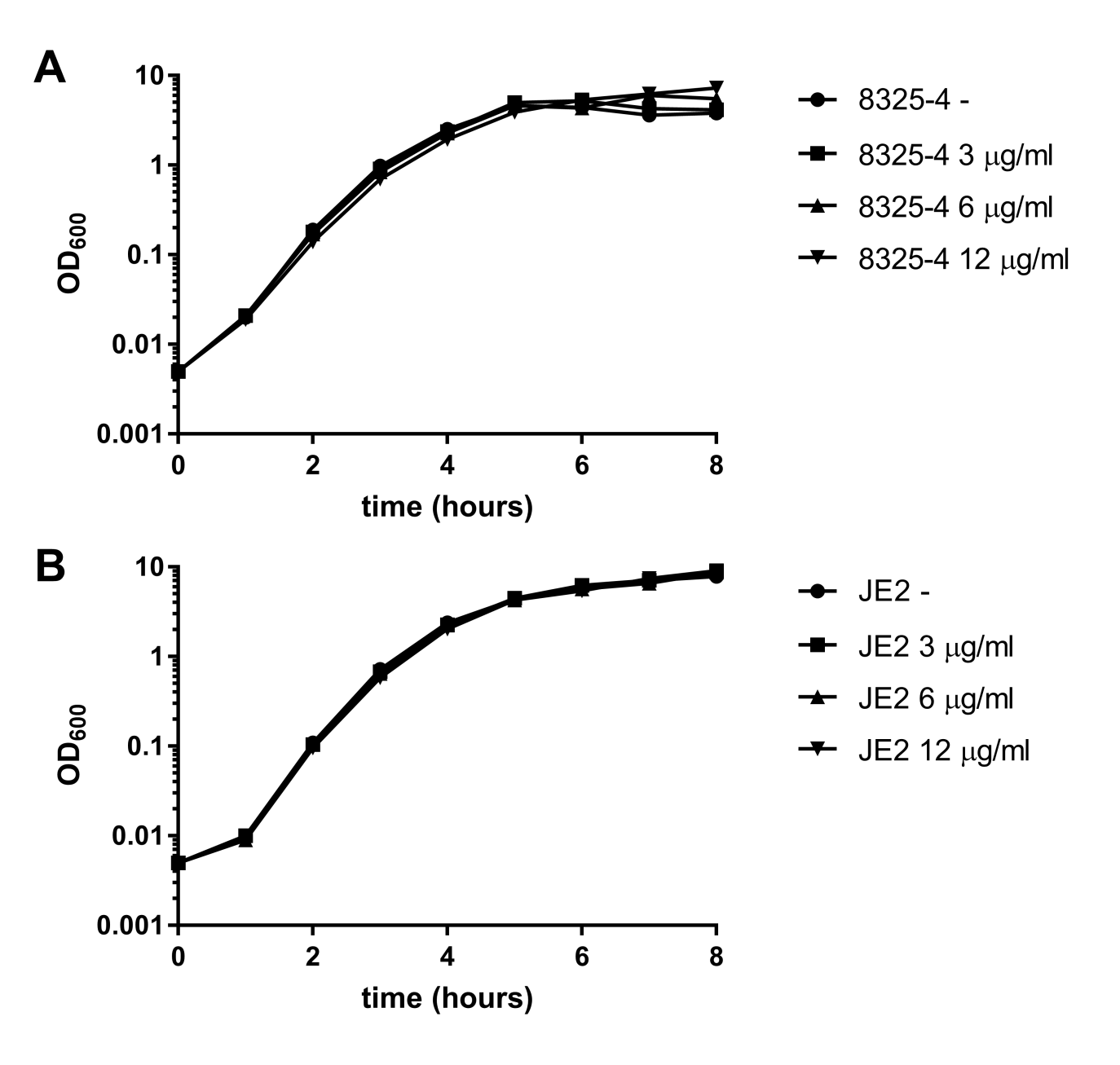


**Supplementary Figure S3: G3 has no notable effect on *S. aureus* growth**. *S aureus* strains 8325-4 (A) or JE2 (B) were inoculated from overnight cultures into fresh TSB to an OD600 of 0.005 with or without peptidomimetic G3 added and incubated at 37ºC in shaking flasks (200 rpm.). OD of the cultures was monitored until stationary phase entrance. Three different concentrations of G3 were used (3μg/ml, 6μg/ml and 12μg/ml). The results are presented in log scale and they confirm that the interference with the *agr* system is not due to growth impairment.

**Supplementary Figure S4**





**Supplementary Figure S4: Hypothesis of how our peptidomimetics can obtain a competitive activity with *S. aureus* AIP-I.** (A) Chemical structure of *S. aureus* AIP-I and (B) a general chemical structure of the most active identified peptidomimetics where positions three and seven can only different in order to mimic the structure of the AIP-I in case that they can adopt a pseudo-macrocyclic arrangement.
